# VELYS robotic‐assisted total knee arthroplasty: Enhanced accuracy and comparable early outcomes versus manual instrumentation during adoption

**DOI:** 10.1002/jeo2.70163

**Published:** 2025-02-10

**Authors:** Timothy B. Alton, Erik P. Severson, Marcus C. Ford, James Lesko, Ian J. Leslie

**Affiliations:** ^1^ Proliance Orthopedic Associates Renton Washington USA; ^2^ Minnesota Center for Orthopaedics Crosby Minnesota USA; ^3^ Campbell Clinic Germantown Tennessee USA; ^4^ DePuy Synthes Warsaw Indiana USA; ^5^ DePuy Synthes Leeds UK

**Keywords:** accuracy, comparative, level II, prospective, robotic‐assisted, VELYS

## Abstract

**Purpose:**

This study assessed the accuracy and early clinical outcomes of the VELYS™ Robotic‐Assisted solution for total knee arthroplasty (TKA).

**Methods:**

A multicenter, prospective non‐randomized 1:1 cohort study was conducted at five sites. Subjects underwent TKA with either manual instrumentation or with robotic‐assistance (RA). RA procedures were the first conducted at each site, therefore, representing the adoption phase for each surgeon. Mechanical alignment was targeted in the manual arm, while the target and technique varied in the RA arm. The primary objective was a non‐inferiority (NI) analysis of the accuracy of the hip–knee–ankle (HKA) for RA versus manual, with a 1.5° NI margin. The accuracy of the mechanical medial distal femoral angle (mMDFA), mechanical medial proximal tibial angle (mMPTA) tibial posterior slope (TPS) angles were measured. Adverse events (AEs) and patient‐reported outcome measures (PROMs) were collected at 12 weeks and 1 year.

**Results:**

One hundred participants were recruited for both manual and RA groups, the mean preoperative demographics and PROM scores were similar. The primary endpoint NI analysis was successful (*p* < 0.0001). The RA group demonstrated improved alignment accuracy of the femoral and tibial components compared to manual (mMDFA 1.3 vs. 1.9, *p* = 0.0026, mMPTA 1.2 vs. 1.5, *p* = 0.026, TPS 1.7 vs. 2.8, *p* < 0.0001). Serious AEs occurred in fewer RA subjects than in the manual (6 vs. 16, *p* = 0.040). Mean PROMs at 12 weeks and 1 year in the RA group compared to manual were either equivalent or improved (Forgotten Joint Score and pain at 12 weeks).

**Conclusions:**

This study found that the RA system can be safely adopted without adversely impacting the long leg alignment or increasing the risk of complications. Further, it was observed that the accuracy of the femoral and tibial component positioning was improved, and there were positive trends in the rate of serious AEs and some PROMs at early follow‐up.

**Level of Evidence:**

Level II.

**Abbreviations**
AE: adverse event
aHKA: arithmetic hip–knee–ankle
AP: anterior‐posterior
BMI: body mass index
CPAK: Coronal Plane Alignment of the Knee
CR: cruciate retaining
FB: fixed bearing
FJS: Forgotten Joint Score
HKA: hip–knee–ankle
JLO: joint line obliquity
KA: kinematic alignment
LCL: lateral collateral ligament
MA: mechanical alignment
MCL: medial collateral ligament
mLDFA: mechanical lateral distal femoral angle
mMDFA: mechanical medial distal femoral angle
mMPTA: mechanical medial proximal tibial angle
PCL: posterior cruciate ligament
PROM: patient‐reported outcome measure
PS: posterior stabilized
PSA: patient‐specific alignment
RA: robotic‐assistance
RMSE: root mean square error
ROM: range of motion
RP: rotating platform
SAE: serious adverse event
TKA: total knee arthroplasty
TPS: tibial posterior slope
VRAS: VELYS Robotic‐Assisted Solution

## INTRODUCTION

Robotic‐assisted surgery for total knee arthroplasty (TKA) has been gaining popularity over the past decade driven by improved accuracy and the insights provided by the wealth of intraoperative data [31]. The VELYS™ Robotic‐Assisted Solution (VRAS) (DePuy Synthes) has recently been introduced. This image‐free system registers patients' landmarks intraoperatively to facilitate implant position planning and guides a handheld saw to the planned cut. This system is currently the only image‐free system that directly provides guidance to a saw handpiece.

While the accuracy of robotic‐assisted systems for TKA has been widely studied, the quality of these assessments is variable [20] and it remains important to assess new systems as they enter the market. Therefore, the primary goal of this study is to evaluate the accuracy of VRAS in a clinical setting, building upon previous cadaveric assessments of accuracy [7].

With the adoption of new technology, there is a learning phase. This can result in increased risk to patients as the surgeon and supporting staff familiarize themselves with the new technique and technology. This study aimed to assess the safety of the system during the learning phase by capturing all adverse events (AEs) specifically during the first clinical uses of the system for the participating surgeons.

The literature on the influence of robotic‐assisted systems on the clinical outcomes of TKA is inconsistent, but some studies have reported advantageous early patient‐reported outcomes (PROMs) [1, 5, 12, 29]. Therefore, tertiary end points were included to assess the impact of VRAS on PROMs at 12 weeks and 1 year post‐operatively.

## METHODS

### Study design

A Level II, multicentre, prospective non‐randomized 1:1 cohort study was conducted at five sites (NCT04730271). Subjects underwent TKA with either manual instruments or robotic‐assistance (RA). RA procedures were the first conducted at each site, therefore, representing the adoption phase. To ensure appropriate capture of learning curve cases the participating surgeons were only permitted to use the VRAS system on study subjects allocated to the RA arm.

Prior to the study initiation, the five participating investigators had experience implanting the ATTUNE™ primary knee system but were not using RA or navigation as part of their standard of care. Two of the participating investigators had prior experience with the MAKO system (Stryker) for TKA during surgical training (<10 cases), one had experience using MAKO for Unicondylar Knees (10–50 cases), and two had no prior clinical robotic experience. Prior to the first cases, all investigators underwent cadaveric training on the VRAS system.

The setting of each investigator varied. One operated exclusively out of an ambulatory service centre, while the others were based in hospitals with access to both inpatient and outpatient departments.

Inclusion criteria included patients aged 22–85 years old with osteoarthritis, posttraumatic arthritis or rheumatoid arthritis and a suitable candidate for TKA. Patients were excluded if they were pregnant, their contra‐lateral knee was already enroled in the study, or they had an amputation, previous partial knee arthroplasty, patellectomy, high tibial osteotomy or primary TKA in the affected knee.

All five participating sites had the initial target of recruiting 20 subjects into each arm, however, cohort reallocation was allowed if sites were not able to meet this target within the study timelines.

Following recruitment, demographics, relevant medical history, pre‐operative radiographs (long Leg AP and standard lateral) and baseline PROMs were collected. Data were collected intraoperatively, subjects were then followed up at 12 weeks and 1 year post‐operatively.

### Procedural information

For the control arm, participating surgeons performed TKA with manual instrumentation using an extramedullary tibial jig and an intramedullary femoral jig for alignment of the primary resections. As per the standard of care of the participating surgeons, neutral mechanical alignment (MA) of the tibia, femur and hip–knee–ankle (HKA) was targeted for manual procedures with the planned angles recorded intraoperatively. For the RA arm VRAS was utilized. VRAS is a semi‐active system that positions the sawblade in the plane of the planned resection, making active adjustments to account for movements of the leg during the resection without the need for cutting blocks. The user is in control of the system within the plane of resection. If the user forces the sawblade outside of the planned resection plane, the power to the saw is cut‐off. When using VRAS, following landmark registration, the plan is created whereby the position of the implants can be adjusted with the resultant impact on the medial and lateral gaps throughout the range of motion displayed. The way the surgeons used this information varied based on surgical philosophy and was not dictated by the protocol; however, the protocol did require that the planned HKA be within ±3° as per the recommended technique for the ATTUNE™ primary system. The planned angles were recorded intraoperatively and verified with comparison to the data captured by the system.

Following the execution of bone resections to check for any iatrogenic damage the condition and function of the medial collateral ligament (MCL), lateral collateral ligament (LCL) posterior cruciate ligament (PCL) (cruciate retaining [CR] cases only), patella tendon, posterior lateral capsule and the posterior medial capsule were assessed and documented.

One of the available constructs from the ATTUNE primary total knee system was implanted according to each participating site's standard of care.

Surgery time was recorded as the time between incision and wound closure, and operating room utilization time was recorded as the time elapsed between the patient entering and then leaving the operating room.

### Radiographic analysis

The primary objective was a non‐inferiority (NI) analysis of the accuracy of the HKA for RA versus manual. Accuracy was defined as the absolute difference of the actual vs. planned HKA measured via post‐operative long leg anterior‐posterior (AP) radiographs captured at the 12‐week visit. The HKA was defined as the angle between the mechanical axis of the femur and the mechanical axis of the tibia [6]. All radiographic analysis was conducted by an independent third party (Medical Metrics Inc.), who were blinded to the treatment group.

**Figure 1:**
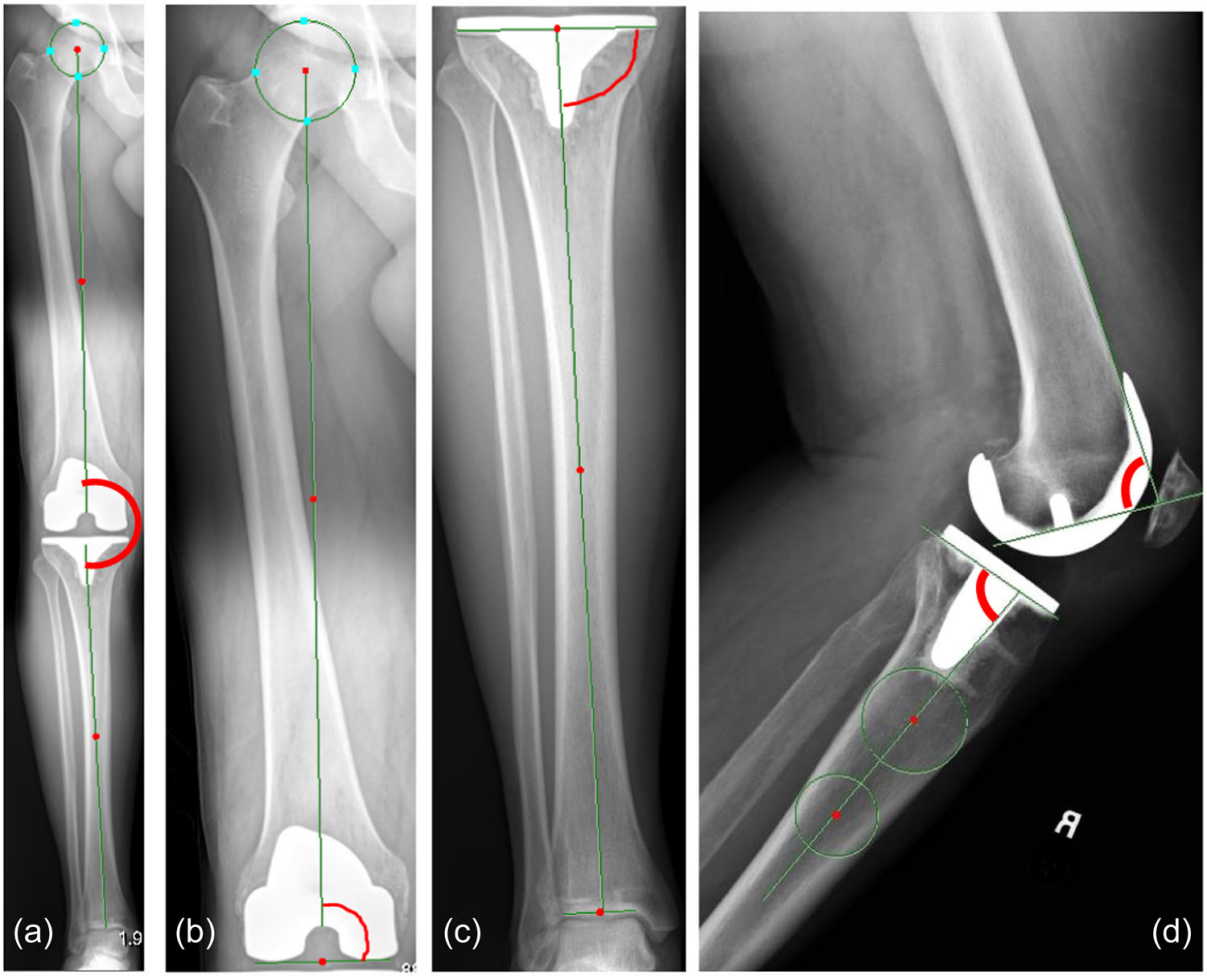
Example x‐ray images with definition of measurements made. (a) Hip–knee–angle measured by taking the angle between the mechanical axis of the femur and the mechanical axis of the tibia. (B) Mechanical medial distal femoral angle. (C) Mechanical medial proximal tibial angle. (D) Tibial posterior slope and femoral flexion. The femoral flexion and posterior tibial slope were measured on a standard view lateral radiograph, this did not allow direct measurement of the angle relative to the mechanical axis.

In addition to the radiographic HKA, the accuracy of the angles of the femoral and tibial implants were assessed, including mechanical medial distal femoral angle (mMDFA), mechanical medial proximal tibial angle (mMPTA), femoral flexion angle and tibial posterior slope (TPS) angles. These were measured radiographically and compared to the planned angles (Figure 1). Accuracy was defined as the absolute difference between the planned angle and the measured angle, reported as the root mean square error (RMSE).

The posterior tibial slope was measured on standard view lateral radiographs, this did not allow direct measurement relative to the mechanical axis, rather the angle between the proximal anatomic axis of the tibia and a line tangent to the top of the tibial baseplate was taken (Figure 1).

Following conclusion of the study, the preoperative and post‐operative Coronal Plane Alignment of the Knee (CPAK) classification was determined by converting the mMDFA to the mechanical lateral distal femoral angle (mLDFA), and subsequently calculating the arithmetic HKA (aHKA) and joint line obliquity (JLO) [14].

### Patient‐reported outcomes

Knee Injury and Osteoarthritis Outcome Score (KOOS), 5‐level EQ‐5D version (EQ‐5D), EuroQol Visual Analogue Scale (EQ VAS), pain while resting, pain with activity were collected preoperatively, and at the 12‐week and 1‐year post‐operative visits. The Forgotten Joint Score (FJS) and satisfaction with the procedure were collected at the 12‐week and 1‐year post‐operative visits.

### Safety information

AEs were collected as they occurred throughout the study. Subjects' medical records were reviewed at the 12‐week and 1‐year follow‐up visits to check for any non‐reported events. AEs were classified as systematic or local, with a local event defined as one occurring at the site of the procedure, as well as serious adverse events (SAEs) (required medical or surgical intervention) or not.

### Statistical methods

The primary endpoint analysis was an NI comparison of the mean of the absolute values of deviation from planned HKA with an NI margin of 1.5° conducted on the intention‐to‐treat (ITT) cohort. The ITT analysis group was defined as any procedure that was initiated with the specified instrumentation system. The NI margin was based upon prior studies that have suggested that a deviation from target of more than 3° may have a detrimental impact on clinical outcomes [21]; thus, half of this margin was deemed to be clinically relevant. The absolute deviation from plan was calculated by calculating the absolute value of the difference between the planned and measured angle values.

Based on the anticipated standard deviation of approximately 2.4° in deviation from plan from prior studies [2], it was estimated that there was greater than 99% power to demonstrate NI in this primary end point analysis with a sample size of *N* = 100 in each group. The sample size of *N* = 100 was also established to provide sufficient data for the evaluation of the nature, severity, and frequency of AEs associated with the use of VRAS during the adoption phase.

Continuous variables were assessed with a *t* test. For ordinal variable comparisons, the Mantel–Haenszel test with 1° of freedom was utilized. For other categorical variable comparisons, a Fisher exact test was utilized.

### Ethical aspects

Prior to initiation, the study received institutional review board (IRB) approval, including from the central IRB (WCG IRB) and local IRB (Lifebridge Health, The University of Tennessee Health Science Center, Novant Health). All patients provided formal informed consent prior to enrolment into the study per Good Clinical Practice.

## RESULTS

### Patients

A total of 200 subjects were enroled and received the planned treatment; 100 in the manual arm and 100 in the RA arm. Eleven subjects from the RA arm were excluded from the per protocol (PP) analysis as the surgeons' intraoperative planned alignment was outside of the ±3° of neutral specified in the protocol. For one subject (1%), the procedure was initiated with the VRAS system but not completed due to the system becoming non‐responsive following registration resulting in the procedure being completed using manual instrumentation. The analysis of end points in both ITT and PP groups was conducted.

Most subjects had a primary diagnosis of osteoarthritis for both manual and RA groups (Table 1). The mean age and gender were similar across groups; the manual group had a higher body mass index than the RA group (33.5 vs. 31.7; *p* = 0.025), although the range was similar. The mean preoperative KOOS, EQ‐5D, Pain and ROM were similar between the groups (Table 1).

**Table 1:** Subject demographics and preoperative patient‐reported outcomes.

	Robotic‐assisted mean (SD)	Manual mean (SD)	*p*
Age at consent	66.6 (8.28)	64.4 (9.05)	0.0771
Sex, *n* (%) female	48	53	0.5717
Primary diagnosis, *n* a			
Osteoarthritis	100	98	0.4975
Rheumatoid arthritis	2	5	0.4448
Post‐traumatic arthritis	1	0	1.000
Body mass index (kg/m^2^)	31.7 (5.45)	33.5 (5.42)	**0.0250**
Body mass index range (min, max)	21.87–44.69	24.31–43.67	N/A
KOOS ADL	46.1 (17.03)	45.3 (17.64)	0.9031
KOOS pain	42.0 (15.40)	38.7 (17.24)	0.3050
KOOS symptoms	47.3 (18.46)	42.7 (17.53)	0.3054
KOOS Sports & Rec	21.5 (21.96)	16.1 (19.47)	0.0655
KOOS quality of life	22.0 (16.45)	18.8 (16.33)	0.3814
EQ‐5D‐5L	0.62 (0.161)	0.61 (0.163)	0.9309
EQ VAS	70.7 (15.22)	72.8 (15.15)	0.3909
Pain Catastrophizing Scale	19.5 (13.49)	17.1 (13.01)	0.2787
Pain at rest (scale 0–10 high)	5.7 (2.50)	6.3 (2.65)	0.1370
Pain with activity (scale 0–10 high)	7.3 (1.99)	7.6 (1.99)	0.1755
Range of motion	109.0 (16.28)	105.2 (17.78)	0.1165

*Note*: Bold indicates statistically significant at *p*‐value below 0.05.

Abbreviations: ADL, activity of daily living; EQ‐5D‐5L, 5‐level EQ‐5D version; EQ VAS, EuroQol Visual Analogue Scale; KOOS, Knee Injury and Osteoarthritis Outcome Score; SD, standard deviation.

^a^ Multiple primary diagnoses are allowed.

### Intraoperative data

The RA cases were completed without any instances of iatrogenic damage to the key soft tissue structures or other intraoperative AEs. Proportionally, more CR implants were used in the RA cohort than in the manual cohort, where more (posterior stabilized) PS implants were used (Table 2). The RA group had increased mean surgical time compared to manual; however, the later RA cases were significantly faster compared to the first 10 (98.8 min vs. 109.1 min; *p* = 0.0327) (Table 2). Although all surgeons targeted neutral MA for every subject in the control arm, there was variation in the targeted HKA for the RA group and the boundaries employed were not consistent between the participating surgeons (Table 3). Further, there was variation in the sequence of the resections, the alignment philosophy and how the gap information was used between the contributing surgeons in the RA arm (Table 3).

**Table 2:** Operative details.

		Robotic‐assisted	Manual	*p*
Setting of care, *N* (%)	Hospital inpatient	37 (37.0)	39 (39.0)	0.9620
	Hospital outpatient	48 (48.0)	47 (47.0)	
	ASC	15 (15.0)	14 (14.0)	
TKA configuration, *N* (%)	CR FB	42 (42.0)	35 (35.0)	**<0.0001**
	CR RP	20 (20.0)	8 (8.0)	
	PS FB	31 (31.0)	25 (25.0)	
	PS RP	7 (7.0)	32 (32.0)	
Planned HKA angle	Mean (SD)	1.3 (2.04)	0.1 (0.37)	**<0.0001**
	Range	−4 to 6.5	0–3	
Planned femur V/V	Mean (SD)	0.3 (1.57)	0.6 (1.57)	0.1273
	Range	−4 to 5	0–6	
Planned tibia V/V	Mean (SD)	1.1 (1.51)	0.0 (0.00)	**<0.0001**
	Range	−2 to 6.5	0–0	
MCL condition	Fully intact	100 (100.0)	98 (98.0)	0.4975
	Partial cleavage	0	2 (2.0)	
LCL condition	Fully intact	100 (100.0)	100 (100.0)	N/A
PCL condition (CR only)	Fully intact	62 (100.0)	28 (93.3)	0.1062
	Partial cleavage	0	1 (3.3)	
	Full cleavage	0	1 (3.3)	
Posterior lateral capsule	Uncompromised	100 (100.0)	100 (100.0)	N/A
Posterior medial capsule	Uncompromised	100 (100.0)	100 (100.0)	N/A
Patella tendon function	Uncompromised	100 (100.0)	100 (100.0)	N/A
Patella tendon condition	Fully intact	100 (100.0)	100 (100.0)	N/A
Surgery time, skin‐skin (min)	All mean (SD)	106.6 (23.56)	87.5 (21.96)	**<0.0001**
	First 10 cases	109.1 (23.19)	N/A	
	Latter cases	98.8 (20.86)	N/A	
OR time, wheels in‐wheels out.	Mean (SD)	159.3 (31.67)	131.6 (28.53)	**<0.0001**

*Note*: Bold indicate statistically significant at *p*‐values below 0.05.

Abbreviations: ASC, ambulatory service centre; CR, cruciate retaining; FB, fixed bearing; HKA, hip–knee–angle; MCL, medial collateral ligament; PCL, posterior cruciate ligament; PS, posterior stabilized; RP, rotating platform; SD, standard deviation; TKA, total knee arthroplasty.

**Table 3:** Summary of surgical technique employed in RA arm with VRAS.

Site number	High level	Cut sequence	HKA boundaries	How gap information used	Subjects (%)	Subjects per protocol^a^
01	MA	Tibia first	±1°	Adjust cuts within boundaries to balance gaps	7 (7%)	7
02	PSA	Tibia first	±2°	Adjust cuts within boundaries to balance gaps	21 (21%)	21
03	Modified MA	Femur first	±5°	Adjust cuts within boundaries to balance gaps	25 (25%)	15
04	Modified MA	Femur first	±2°	Adjust cuts within boundaries to balance gaps	32 (32%)	30
05	KA	Femur first	±5°	Ignore gap information during planning phase	15 (15%)	13

*Note*: Bold indicates statistically significant at *p*‐value below 0.05.

Abbreviations: HKA, hip–knee–angle; MA, mechanical alignment; PSA, patient‐specific alignment; RA, robotic‐assistance; VRAS, VELYS Robotic‐Assisted Solution.

^a^ Per protocol indicates subjects where the surgical target HKA was within the protocol defined ±3° and the 12‐week follow‐up visit was complete.

### Accuracy

**Figure 2:**
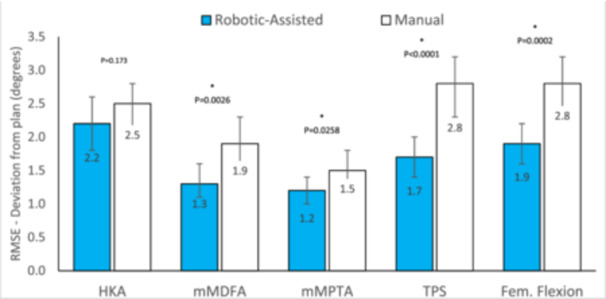
Summary of accuracy results. Error bars—95% Confidence limits. Results presented for the intention‐to‐treat (ITT) study population, which includes 11 subjects in the RA arm excluded from PP due to the planned HKA being outside ±3°. Findings of analysis of per protocol analysis set were consistent with findings with ITT (see Supporting Information S1: Data). HKA, hip–knee–ankle; mMDFA, mechanical medial distal femoral angle; mMPTA, mechanical medial proximal tibial angle; PP, per protocol; RA, robotic‐assistance; RMSE, root mean square error; TPS, tibial posterior slope.

Within the ITT analysis group, the HKA accuracy was non‐inferior in the RA group compared to manual control under the 1.5° NI margin (*p* < 0.0001). There was no significant difference in the mean absolute error of the HKA between the RA and manual group (2.2 vs. 2.5, *p* = 0.173). The same was true if the per protocol analysis set was used (2.3 vs. 2.5, *p* = 0.284). While the accuracy of the HKA was non‐inferior between the two cohorts, the accuracy of the individual femoral and tibial implant angles was significantly improved in the RA group. Specifically, the RA group was associated with improved accuracy of the mMDFA (1.3 vs. 1.9, *p* = 0.0026), mMPTA (1.2 vs. 1.5, *p* = 0.0258), femoral flexion (1.9 vs. 2.8, *p* = 0.0002) and TPS (1.7 vs. 2.8, *p* < 0.0001) (Figure 2). The results and findings were consistent between the PP and ITT analysis groups.

### Safety information

A total of 29 SAEs were reported during the study that required medical or surgical intervention, of these 7 were in the RA group and 22 in the manual control group (Table 4). There was a statistically significant reduction in the number of subjects that experienced an SAE in the RA group compared to the manual group (6 vs. 16, *p* = 0.040). All seven SAEs in the RA group were for arthrofibrosis that occurred in six subjects who were treated with manipulations under anaesthesia (MUAs). In the manual control group, there were nine occurrences of arthrofibrosis with MUAs, nine infections in five subjects with wash out, irrigation, and insert swap outs, two tendon injuries, one arthrotomy disruption that required surgical repair and one revision of the tibial tray for loosening (Table 4).

**Table 4:** Summary of local adverse events.

	Robotic‐assisted		Manual		RA vs. Manual
	Events	Subjects	Events	Subjects	*p* b
Serious adverse events^a^	7	6	22	16	**0.040**
Arthrofibrosis resulting in MUA	7	6	9	8	0.783
Infection	0	0	9	5	0.059
Tendon injury	0	0	2	2	0.497
Arthrotomy disruption	0	0	1	1	1
Tibial loosening	0	0	1	1	1

*Note*: Analysis conducted on intention to treat cohort. Bold indicates statistically significant at *p*‐value below 0.05.

Abbreviations: MUA, manipulation under anaesthesia; RA, robotic‐assistance.

^a^ Serious adverse event defined as an event that requires medical or surgical intervention.

^b^ Fisher's exact test proportion of subjects that experienced an event.

### Patient‐reported outcomes

Subjects from both groups showed significant improvement in mean values for all PROMs from preoperative baseline to 12 weeks (*p* < 0.0001) and to 1 year (*p* < 0.0001). The mean scores were similar between the RA and Manual groups for KOOS, EQ‐5D, EQ VAS, Pain during activity and satisfaction. However, the RA group showed significant improvement in FJS (35.4 vs. 26.4, *p* = 0.0129) and Pain (2.3 vs. 3.4, *p* = 0.0045) at 12 weeks (Table 5). The results and conclusions were consistent if the analysis was conducted on the PP or the ITT analysis groups with the exception of pain during activity at 12 weeks, where the *p* value increases to 0.079 in the PP group versus 0.0364 in the ITT.

**Table 5:** Summary of patient‐reported outcome measures.

Outcome measure	Time point	Robotic mean (SD) ITT^a^	Manual mean (SD) ITT^a^	*p* Manual vs. RA ITT^a^
FJS	12 weeks	35.4 (25.57)	26.4 (23.26)	**0.0129**
	1 year	51.8 (29.81)	45.8 (33.57)	0.2233
KOOS ADL	12 weeks	72.3 (19.12)	69.5 (18.67)	0.3027
	1 year	82.3 (17.70)	77.9 (20.21)	0.1350
	CFB to 1 year	36.5 (19.83)	29.4 (22.92)	0.0933
KOOS Pain	12 weeks	67.4 (19.88)	63.7 (19.26)	0.1991
	1 year	79.3 (19.01)	75.4 (20.76)	0.2083
	CFB to 1 year	37.8 (21.88)	35.4 (23.94)	0.4984
KOOS Symptoms	12 weeks	68.2 (16.97)	64.5 (17.32)	0.1424
	1 year	76.5 (16.42)	72.5 (17.67)	0.1283
	CFB to 1 year	30.6 (21.36)	28.2 (23.79)	0.4968
KOOS Sports & Rec	12 weeks	46.4 (31.59)	40.6 (30.15)	0.2114
	1 year	57.9 (28.08)	56.3 (32.16)	0.7415
	CFB to 1 year	40.0 (31.33)	37.1 (35.21)	0.5917
KOOS QoL	12 weeks	55.8 (24.05)	50.7 (22.70)	0.1378
	1 year	67.9 (23.18)	62.1 (25.93)	0.1318
	CFB to 1 year	45.9 (25.43)	42.0 (31.12)	0.3757
EQ‐5D‐5L	12 weeks	0.78 (0.129)	0.77 (0.143)	0.4052
	1 year	0.83 (0.148)	0.82 (0.129)	0.7598
	CFB to 1 year	0.20 (0.170)	0.20 (0.192)	0.9936
EQ VAS	12 weeks	79.3 (13.14)	77.5 (14.55)	0.3543
	1 year	82.4 (12.05)	79.8 (12.82)	0.1692
	CFB to 1 year	9.7 (13.87)	7.9 (17.97)	0.4649
Pain at rest^b^	12 weeks	2.3 (2.36)	3.4 (2.77)	**0.0045**
	1 year	1.5 (2.24)	2.1 (2.52)	0.1108
	CFB to 1 year	−4.1 (2.93)	−4.0 (2.69)	0.8179
Pain during activity^b^	12 weeks	2.9 (2.38)	3.7 (2.87)	**0.0364**
	1 year	2.1 (2.47)	2.6 (2.57)	0.2229
	CFB to 1 year	−5.2 (2.79)	−5.0 (2.67)	0.7527
Satisfaction^c^	12 weeks	1.4 (2.11)	1.7 (2.44)	0.4739
	1 year	1.6 (2.79)	1.4 (2.46)	0.7155
Range of motion	12 weeks	115.8 (11.22)	112.3 (16.14)	0.0851
	CFB to 12 weeks	7.2 (19.88)	7.2 (21.97)	0.9823

*Note*: Bold indicate statistically significant at *p*‐values below 0.05.

Abbreviations: ADL, activity of daily living; CFB, change from baseline; EQ‐5D‐5L, 5‐level EQ‐5D version; EQ VAS, EuroQol Visual Analogue Scale; FJS, Forgotten Joint Score; KOOS, Knee Injury and Osteoarthritis Outcome Score; QoL, quality of life; RA, robotic‐assistance; SD, standard deviation.

^a^ ITT—Intention‐to‐treat cohort which includes the 11 subjects excluded from RA per protocol cohort due to the planned HKA being outside of ±3°. If cohort is limited to per protocol, results and findings are consistent (see Supporting Information S1: Data) with exception of pain during activity at 12 weeks, where the *p* value increases to 0.079.

^b^ Pain measured on a 10 point scale with 0 *low* and 10 *high*.

^c^ Satisfaction measured on a 10‐point scale with 0 *fully satisfied* and 10 *fully unsatisfied*.

### CPAK classification

**Figure 3:**
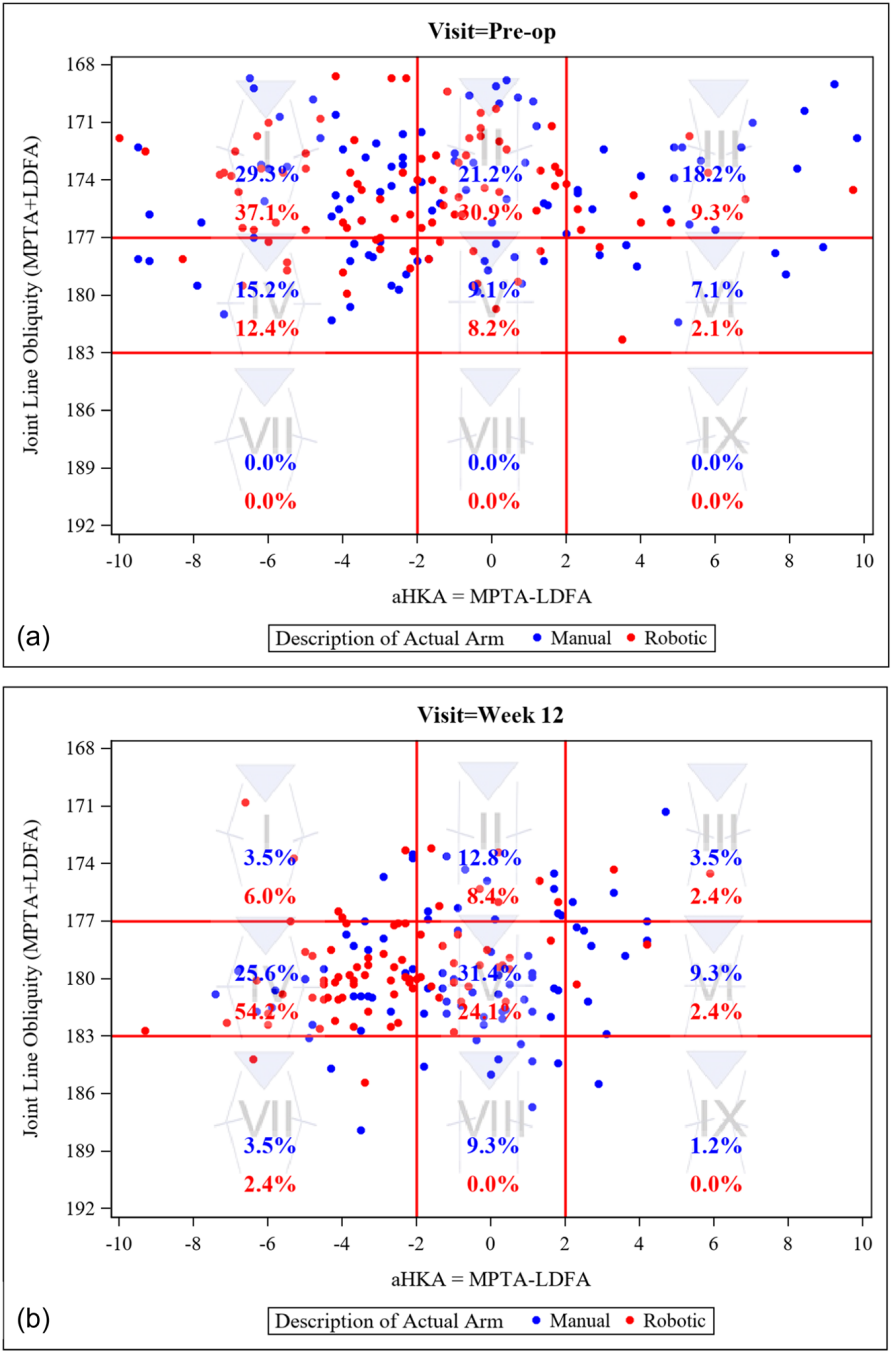
CPAK plots of manual and RA groups pre‐op (a) and post‐op (b). aHKA, arithmetic hip–knee–ankle; CPAK, Coronal Plane Alignment of the Knee; LDFA, lateral distal femoral angle; MPTA, medial proximal tibial angle; RA, robotic‐assistance.

For both groups, the most common preoperative CPAK classification was I (29.3% for manual and 37.1% for RA), followed by II (21.2% for manual and 30.9% for RA) (Figure 3). Post‐operatively, the most common CPAK classification was V for manual (31.4%) and IV for the RA group (54.2%) (Figure 3).

## DISCUSSION

The most important finding of the study was that the VRAS system for TKA can be safely adopted without adversely impacting the leg alignment or rate of AEs. This study is the first multisite prospective clinical study to report on the accuracy and early clinical outcomes of the VRAS system. The observed improvement in the accuracy of the angles of the femoral and tibial components correlates well with the findings of a prior cadaveric study on the VRAS system [7]. A recent retrospective clinical study found reduced outliers in LDFA and tibial slope for VRAS compared to manual [19]. These findings also correlate well with results from cadaveric and clinical studies on other RA systems which also broadly show improvements in accuracy in implant positioning compared to manual instrumentation [13, 15, 18, 26, 28, 30]. However, direct comparisons between studies are challenging due to significant differences in measurement methodologies, and definitions of accuracy [20]. The use of a method independent of the RA system to assess the accuracy in this study (x‐ray analysis), encompassing all potential sources of error is a strength of the study design. Further, this study included five different surgeons and no cases were excluded from the analysis to allow for a learning curve making this study a rigorous assessment of the system.

Although the accuracy of the system in the sagittal plane was found to be superior to manual instrumentation, the average errors were higher than in the coronal plane. This is consistent with the findings on other systems [3, 20, 23] and may be related to the repeatability of the registration within the coronal plane; conversely, it may be associated with a limitation in the study design as only standard view lateral x‐rays were captured which do not allow the direct measurement of the mechanical axis which is defined and used as the reference in the planning of the VRAS system.

The study also demonstrated that the HKA was non‐inferior to manual instrumentation. However, with the significant improvement in the accuracy of the femoral varus/valgus and tibial varus/valgus angles for RA, a similar improvement in the accuracy of HKA would also be anticipated, but this was not observed. There are many potential reasons for this. The planned HKA for the RA group was determined by assessing the subject in the supine position whilst the radiographic review was completed with the patient's weight bearing. A prior study has found significant differences between supine HKA and the standing radiographic HKA [22]. When assessing the accuracy of the HKA in a 2D x‐ray, there can be errors introduced by leg rotation [27]. Errors caused by leg rotation can be further magnified when the leg is in varus [17]; therefore, there may have been a penalizing effect for the RA group which, unlike the manual instrument arm, on average targeted a varus HKA.

The pre‐operative CPAK classifications and distributions are consistent with previous publications [14, 25]. The lack of apex proximal knees (types VII, VIII and IX) and the lack of ability to account for the magnitude of deformity question the necessity of these categories and may warrant the use of alternative classification systems [9]. The post‐operative CPAK classifications are indicative of the differences in the surgical plans between the manual and RA groups, with the most common classification for the manual group (V) neutral JLO and aHKA and the most common classification for the RA group (IV) having a varus aHKA. Interestingly, there is a general increase in the JLO from pre‐op to post‐op for both groups, suggesting that restoring the angle of the joint line was not of primary consideration in the surgical plan for either group.

The increased surgical time for the RA group was anticipated and associated with the surgeons and surgical teams being on a learning curve associated with adopting the technology. The reduction in mean surgical time between the first 10 and subsequent cases demonstrates this. Other studies on the VRAS system have demonstrated that time neutrality with manual instrumentation can be achieved following a learning curve [16, 24]. However, the number of cases to get to this point will vary between surgeons based on many factors and the surgeons participating in this study may experience further reduction in surgical time with increased experience.

The safety data gathered in this study indicated that the adoption of VRAS was not associated with an increase in adverse events compared to the standard of care manual instrumentation. The study found no significant iatrogenic soft tissue damage in the RA arm, demonstrating that the VRAS system can be used without compromising soft tissue structures. This is pertinent because, while the surgeon has full control of the saw within the resection plane, due to the saw's connection to the planer articulation and adjustments being made by the system to keep the saw in plane, there may be differences in the proprioception compared to using a saw in a manual procedure. Conversely, the lack of cutting blocks may improve the visibility of the saw during resections. The significant reduction in serious adverse events in the RA arm compared to manual is an interesting observation and supports findings of a recent database study with a larger sample size that found VRAS to be associated with a reduced rate of knee‐related revisits versus manual instrumentation [11]. However, the rate of SAEs observed in the manual arm in this study was high relative to what is typically observed in primary TKA, specifically the rate of infections [8]. Given the control arm data was standard of care at the participating sites, the reasons for this are not known and therefore can only be assumed to be a random anomaly. Large‐scale, real‐world evidence studies are required to determine if the observed trend from this study can be confirmed.

Overall, the PROMS were similar between the RA and manual groups with no statistical differences at 1 year, however, broadly the mean PROM scores were trending positively for RA compared to manual and FJS and pain were significantly better at 12 weeks. This is consistent with the findings of other studies that have compared RA with manual [1, 4, 5, 12]. However, these findings need to be confirmed with studies specifically designed to assess these endpoints. The positive trends observed for the RA group could be attributed to changes in techniques between the study arms with some of the participating surgeons utilizing patient‐specific techniques in the RA arm which reduce the need for soft tissue releases [24] and are further confounded by the lack of consistency within the RA arms as the participating surgeons developed their techniques during the adoption phase. A recent study that compared a single surgeon's outcomes using VRAS to Navigation with a consistent surgical technique between the arms found improved surgical efficiency for VRAS but no significant differences in PROMs [10]. This study was non‐randomized and therefore susceptible to bias in both patient selection and patient perception of the procedure. Further, the variability between the postoperative regimes, pain control and rehab practices at the five participating sites also could have influenced the early clinical outcomes reported. Despite these limitations, the primary objectives of the study to assess the accuracy and safety of the system are not influenced by the specific surgical technique employed, and having variation in techniques, it makes the findings more generalizable and relevant to the broader clinical use of the system.

## CONCLUSION

This study has demonstrated that the VELYS Robotic‐Assisted Solution system for TKA can be safely adopted without adversely impacting the leg alignment or rate of adverse events. The accuracy of femoral and tibial component positioning was improved compared to manual instrumentation. Less serious adverse events were observed with RA compared to manual and positive trends were observed in RA for FJS and pain at 12 weeks. These observed differences equalized to no significant differences in PROMs at 1 year. Further studies are required to assess the clinical benefits of this system.

## AUTHOR CONTRIBUTIONS

Conceptualization: Ian J. Leslie and James Lesko. Methodology: Ian J. Leslie, James Lesko, Timothy B. Alton, Erik P. Severson and Marcus C. Ford. Subject Recruitment and Data Capture: Timothy B. Alton, Erik P. Severson and Marcus C. Ford. Formal analysis and investigation: James Lesko. Writing—original draft preparation: Ian J. Leslie. Writing—review and editing: Timothy B. Alton, Erik P. Severson, Marcus C. Ford and James Lesko.

## CONFLICT OF INTEREST STATEMENT

Timothy B. Alton, Erik P. Severson and Marcus C. Ford received institutional research funding to conduct this research. Timothy B. Alton, Erik P. Severson and Marcus C. Ford are consultants to DePuy Synthes. Ian J. Leslie and James Lesko are paid employees of DePuy Synthes.

## ETHICS STATEMENT

Prior to initiation, the study received institutional review board (IRB) approval, including using central IRB (WCG IRB) and local IRB (Lifebridge Health, The University of Tennessee Health Science Center, Novant Health). All patients provided formal informed consent prior to enrolment into the study per Good Clinical Practice.

## Supporting information

Supporting information.

## Data Availability

Data can be requested through the Yale Open Data Access (YODA) project: https://dev-yoda.pantheonsite.io/jj-available-data/.
