# Supplementary material for: VELYS robotic‐assisted total knee arthroplasty: Enhanced accuracy and comparable early outcomes versus manual instrumentation during adoption
Source: J Exp Orthop. 2025 Feb 10;12(1):e70163. doi: 10.1002/jeo2.70163 (PMC11808256; doi:10.1002/jeo2.70163)
Supplement: Supplementary file 1 — Supporting information. [file JEO2-12-e70163-s001.docx]

**Supplementary Data:**

Table 1 and Figure 1 show the results from the analysis of the per protocol (PP) and Intention to treat (ITT) cohorts side by side.

Table 1: Replication of Table 3 from manuscript including PP and ITT data for illustration purposes.

| **Outcome Measure** | **Time Point** | **Robotic**  **Mean (SD)**  **Per protocol** | **Manual**  **Mean (SD)**  **Per Protocol** | **P-value**  **Manual Vs. RA per protocol** | **Robotic Mean (SD) ITT** | **Manual Mean (SD) ITT** | **P-Value Manual Vs. RA ITT** |
| --- | --- | --- | --- | --- | --- | --- | --- |
| FJS | 12 weeks | 35.4 (25.73) | 26.4 (23.39) | **0.0165** | 35.4 (25.57) | 26.4 (23.26) | **0.0129** |
|  | 1 year | 53.1 (28.72) | 45.5 (33.59) | 0.1331 | 51.8 (29.81) | 45.8 (33.57) | 0.2233 |
|  |  |  |  |  |  |  |  |
| KOOS ADL | 12 weeks | 72.0 (19.21) | 69.5 (18.77) | 0.3879 | 72.3 (19.12) | 69.5 (18.67) | 0.3027 |
|  | 1 year | 82.5 (16.90) | 78.0 (20.28) | 0.1396 | 82.3 (17.70) | 77.9 (20.21) | 0.1350 |
|  | CFB to 1 yr. | 35.9 (18.93) | 31.0 (23.21) | 0.1596 | 36.5 (19.83) | 29.4 (22.92) | 0.0933 |
|  |  |  |  |  |  |  |  |
| KOOS Pain | 12 weeks | 67.1 (19.86) | 63.8 (19.33) | 0.2737 | 67.4 (19.88) | 63.7 (19.26) | 0.1991 |
|  | 1 year | 79.4 (18.85) | 75.3 (20.95) | 0.2016 | 79.3 (19.01) | 75.4 (20.76) | 0.2083 |
|  | CFB to 1 yr. | 37.5 (21.68) | 35.3 (24.21) | 0.5675 | 37.8 (21.88) | 35.4 (23.94) | 0.4984 |
|  |  |  |  |  |  |  |  |
| KOOS Symptoms | 12 weeks | 67.8 (17.35) | 64.7 (17.27) | 0.2438 | 68.2 (16.97) | 64.5 (17.32) | 0.1424 |
|  | 1 year | 77.6 (15.92) | 72.6 (17.82) | 0.0686 | 76.5 (16.42) | 72.5 (17.67) | 0.1283 |
|  | CFB to 1 yr. | 31.1 (21.13) | 28.4 (24.02) | 0.4586 | 30.6 (21.36) | 28.2 (23.79) | 0.4968 |
|  |  |  |  |  |  |  |  |
| KOOS Sports & Rec | 12 weeks | 45.0 (30.69) | 39.9 (29.64) | 0.2867 | 46.4 (31.59) | 40.6 (30.15) | 0.2114 |
|  | 1 year | 58.6 (27.35) | 56.8 (32.10) | 0.7131 | 57.9 (28.08) | 56.3 (32.16) | 0.7415 |
|  | CFB to 1 yr. | 39.5 (30.12) | 38.1 (35.09) | 0.8000 | 40.0 (31.33) | 37.1 (35.21) | 0.5917 |
|  |  |  |  |  |  |  |  |
| KOOS QoL | 12 weeks | 55.9 (24.29) | 51.1 (22.47) | 0.1755 | 55.8 (24.05) | 50.7 (22.70) | 0.1378 |
|  | 1 year | 68.8 (21.90) | 62.4 (25.62) | 0.0939 | 67.9 (23.18) | 62.1 (25.93) | 0.1318 |
|  | CFB to 1 yr. | 45.6 (23.67) | 42.3 (31.42) | 0.4636 | 45.9 (25.43) | 42.0 (31.12) | 0.3757 |
|  |  |  |  |  |  |  |  |
| EQ-5D 5L | 12 weeks | 0.78 (0.129) | 0.77 (0.143) | 0.5340 | 0.78 (0.129) | 0.77 (0.143) | 0.4052 |
|  | 1 year | 0.83 (0.151) | 0.82 (0.129) | 0.8346 | 0.83 (0.148) | 0.82 (0.129) | 0.7598 |
|  | CFB to 1 yr. | 0.19 (0.159) | 0.20 (0.195) | 0.6662 | 0.20 (0.170) | 0.20 (0.192) | 0.9936 |
|  |  |  |  |  |  |  |  |
| EQ VAS | 12 weeks | 78.8 (13.47) | 77.8 (14.35) | 0.6129 | 79.3 (13.14) | 77.5 (14.55) | 0.3543 |
|  | 1 year | 82.1 (12.26) | 80.2 (12.52) | 0.3296 | 82.4 (12.05) | 79.8 (12.82) | 0.1692 |
|  | CFB to 1 yr. | 10.2 (13.93) | 7.7 (18.19) | 0.3419 | 9.7 (13.87) | 7.9 (17.97) | 0.4649 |
|  |  |  |  |  |  |  |  |
| Pain at Rest | 12 weeks | 2.3 (2.38) | 3.4 (2.75) | **0.0074** | 2.3 (2.36) | 3.4 (2.77) | **0.0045** |
|  | 1 year | 1.4 (2.14) | 2.1 (2.52) | 0.0663 | 1.5 (2.24) | 2.1 (2.52) | 0.1108 |
|  | CFB to 1 yr. | -4.1 (2.93) | -4.0 (2.71) | 0.8887 | -4.1 (2.93) | -4.0 (2.69) | 0.8179 |
|  |  |  |  |  |  |  |  |
| Pain during activity | 12 weeks | 2.9 (2.43) | 3.6 (2.81) | 0.0709 | 2.9 (2.38) | 3.7 (2.87) | **0.0364** |
|  | 1 year | 1.9 (2.37) | 2.5 (2.58) | 0.1274 | 2.1 (2.47) | 2.6 (2.57) | 0.2229 |
|  | CFB to 1 yr. | -5.2 (2.78) | -5.0 (2.71) | 0.7096 | -5.2 (2.79) | -5.0 (2.67) | 0.7527 |
|  |  |  |  |  |  |  |  |
| Satisfaction | 12 weeks | 1.3 (2.03) | 1.6 (2.29) | 0.4851 | 1.4 (2.11) | 1.7 (2.44) | 0.4739 |
|  | 1 yr. | 1.6 (2.73) | 1.4 (2.37) | 0.6486 | 1.6 (2.79) | 1.4 (2.46) | 0.7155 |
|  |  |  |  |  |  |  |  |
| Range of Motion | 12 weeks | Not Analysed | | | 115.8 (11.22) | 112.3 (16.14) | 0.0851 |
|  | CFB to 12 wks. | N/A | | | 7.2 (19.88) | 7.2 (21.97) | 0.9823 |

Figure 1) Replication of Figure 2 from manuscript including PP and ITT data sets.
